# Supplementary material for: The interactome of intact mitochondria by cross-linking mass spectrometry provides evidence for coexisting respiratory supercomplexes
Source: Mol Cell Proteomics. 2017 Dec 8;17(2):216–32. doi: 10.1074/mcp.RA117.000470 (PMC5795388; doi:10.1074/mcp.RA117.000470)
Supplement: Supplemental Data [file supp_RA117.000470_133922_0_supp_23978_fzffwf.zip › spectra_annotation/mito_DR_spectra_annotation/189-1-3-1-2-1.pdf]

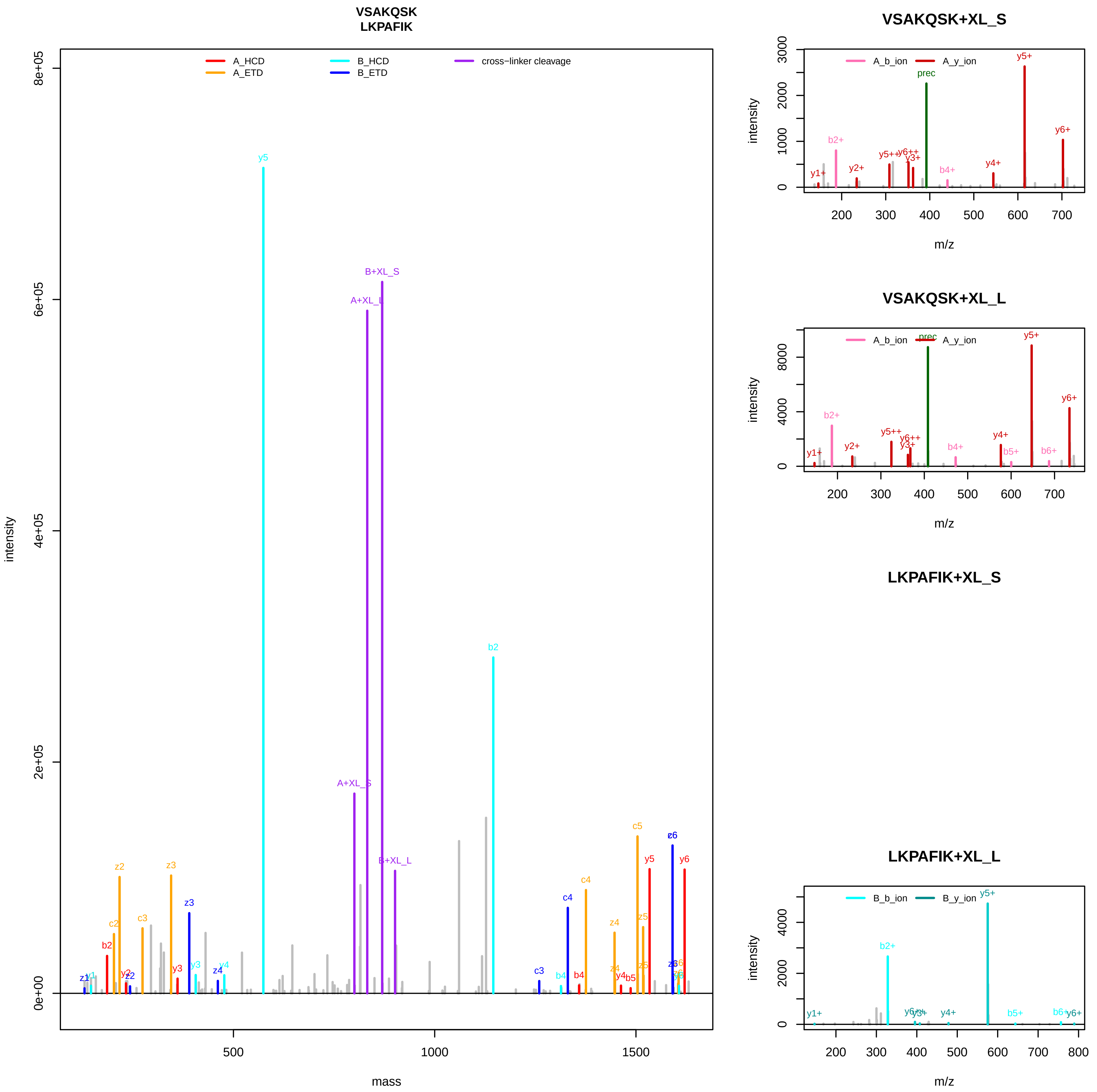

VSAKQSK
LKPAFIK
A_HCD
A_ETD
B_HCD
B_ETD
cross−linker cleavage
intensity
mass
0e+00
2e+05
4e+05
6e+05
8e+05
500
1000
1500
y5
B+XL_S
A+XL_L
b2
A+XL_S
B+XL_L
c5
z6
y5
y6
c4
c3
c2
z2
z3
c3
z3
b2
y3
y3
z4
y4
b4
c4
z4
z5
y4
b5
VSAKQSK+XL_S
A_b_ion
A_y_ion
prec
y5+
y6+
b2+
y5++
y6++
y3+
y2+
y1+
b4+
y4+
m/z
VSAKQSK+XL_L
b5+
b6+
LKPAFIK+XL_S
LKPAFIK+XL_L
B_b_ion
B_y_ion
y1+
y6++
y3+
y4+
b5+
b6+
y6+
